# Supplementary material for: The interactome of intact mitochondria by cross-linking mass spectrometry provides evidence for coexisting respiratory supercomplexes
Source: Mol Cell Proteomics. 2017 Dec 8;17(2):216–32. doi: 10.1074/mcp.RA117.000470 (PMC5795388; doi:10.1074/mcp.RA117.000470)
Supplement: Supplemental Data [file supp_RA117.000470_133922_0_supp_23978_fzffwf.zip › spectra_annotation/mito_DR_spectra_annotation/495-1-10-1-12-1.pdf]

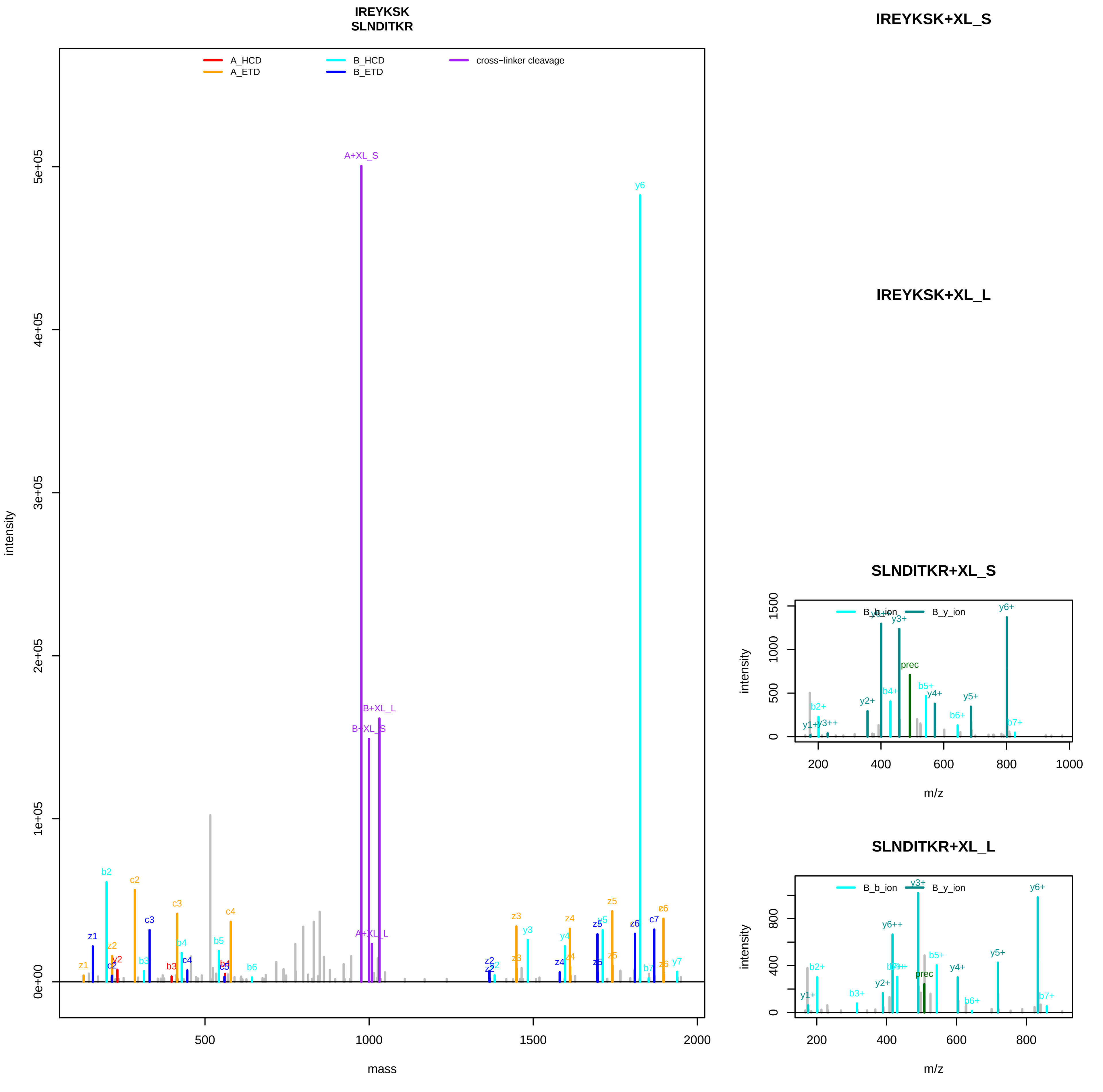

IREYKSK
SLNDITKR
A_HCD
A_ETD
B_HCD
B_ETD
cross−linker cleavage
A+XL_S
y6
B+XL_L
B+XL_S
A+XL_L
b2
c2
c3
c4
z1
z2
y2
c2
b3
c3
b3
b4
c4
b5
b4
c5
b6
z2
z2
y2
z3
z3
y3
z4
z4
y4
z4
z5
y5
z5
z5
z6
z6
c7
b7
z6
c6
y7
0e+00
1e+05
2e+05
3e+05
4e+05
5e+05
intensity
500
1000
1500
2000
mass
IREYKSK+XL_S
IREYKSK+XL_L
SLNDITKR+XL_S
B_b_ion
B_y_ion
y6+
y3+
prec
y2+
b4+
b5+
y4+
y5+
b6+
b2+
y1+
y3++
b7+
0
500
1000
1500
200
400
600
800
1000
m/z
SLNDITKR+XL_L
B_b_ion
B_y_ion
y3+
y6+
y6++
b5+
y5+
y4+
b2+
prec
y2+
b3+
y1+
b6+
b7+
0
400
800
200
400
600
800
m/z
